# Antibiotics use evaluation among hospitalized adult patients at Jimma Medical Center, southwestern Ethiopia: the way to pave for antimicrobial stewardship

**DOI:** 10.1186/s40545-022-00490-4

**Published:** 2022-11-17

**Authors:** Mesay Dechasa, Legese Chelkeba, Amente Jorise, Birbirsa Sefera, Tsegaye Melaku

**Affiliations:** 1 Department of Clinical Pharmacy, School of Pharmacy, College of Health and Medical Sciences, Haramaya University, P.O. Box 235, Harar, Ethiopia; 2 Department of Clinical Pharmacy, School of Pharmacy, Institute of Health Sciences, Jimma University, PO. Box 378, Jimma, Ethiopia; 3 Department of Pharmacy, Clinical Pharmacy Unit, Ambo University, Ambo, Ethiopia; 4 Department of Pharmacy, College of Health Sciences, Mettu University, Mettu, Ethiopia

**Keywords:** Antibiotics, Antibiotic utilization, Antimicrobial Stewardship Programs, Hospitalized patients, Jimma Medical Center, Ethiopia

## Abstract

**Background:**

An irrational antibiotic use is a common problem in developing countries like Ethiopia, which makes empiric antibiotics use difficult. It is considered to be the greatest health problem in our time and future unless intervened. Therefore, this study aimed to assess the patterns of antibiotics use among hospitalized adult patients to pave the way for antimicrobial stewardship.

**Methods:**

A hospital-based prospective observational study was conducted at Jimma Medical Center, southwestern Ethiopia, from 30 October 2020 to 29 January 2021 with 360 adult hospitalized patients participating. A semi-structured questionnaire and consecutive sampling technique was used for data collection. The data were collected through medical record reviews and patient interviews. The collected data were entered into Epi-data and exported to SPSS^®^ version 23.0 for analysis. Days of therapy (DOT) and essential medicine lists “Access, Watch, and Reserve (AWaRe)” antibiotics classification were used to assess antibiotic use pattern among participants.

**Results:**

The majority of study participants were females (55.3%), attended formal education (59.4%), and live in rural areas (61.4%) with mean age ± (SD) of 37.65 ± (16.75). The overall rate of antibiotics consumption during the study was 111 days of therapy per 100 bed-days and about two-thirds (66%) of the prescribed antibiotics were from the “Watch” group antibiotics. The indicator level of antibiotics use for “Access” group antibiotics was 34% in this study based on the World Health Organization Essential Medicine List. Cephalosporins were the most commonly used class of antibiotics (93.9%).

**Conclusion:**

Higher antibiotics exposure and their consumption frequently observed among adult hospitalized patients in the study setting. There was a rapid increase in “Watch” group antibiotics use and about two-thirds of the prescribed antibiotics were from this group. The third-generation cephalosporin were the most commonly used class of antibiotics. Generally, higher consumption and inappropriate antibiotics use among hospitalized adult patients showed the need for urgent interventions by implementing Antimicrobial Stewardship Programs in hospitals.

## Background

Antibiotics are medications used in bacterial infection prevention or/and treatment. Currently, antibiotics are facing global public health threats such as antimicrobial resistance and inappropriate use [1]. According to World Health Organization (WHO), more than two-thirds of antibiotics are used in hospitals and of these antibiotics, about 30% are used inappropriately globally [2].

Excessive antibiotics use may lead to the development of their resistance [3, 4]. There is a stronger link between high antibiotics consumption and their resistance. Globally, increased consumption of antibiotics not only produces greater antibiotics resistance at individual patient, but also at the community level due to irrational antibiotics use [4].

Antimicrobial resistance (AMR) is the greatest global health problem and deserves more attention to ensure effective long-term antibiotics use [5]. The acceleration of AMR and the decline in the development of new antimicrobial to combat a problem has also created significant challenges to health care systems [6]. Inappropriate antibiotics utilization and resistance of antibiotics are increasingly important public health issues based on alarming accumulated facts regarding antibiotics misuse and their resistance in the previous few years [7, 8].

The overall inappropriate antibiotics use is about one-third in low- and middle-income countries (LMICs). Almost 90% of antibiotics prescribed are broad-spectrum agents such as third or fourth-generation Cephalosporins, which need implementation of Antimicrobial Stewardship Programs in hospitals [9]. Rapid increases in “Watch” group antibiotics use, particularly in LMICs, indicate challenges in antimicrobial stewardship [10].

The proportions of irrational antibiotics utilization and their resistance across different countries are varied in Africa. Irrational antibiotics use is about 88.8% in Tanzania [11], 50% in Sudan [8], 7.9% in Zambia [8], and greater than 10% in other African countries [12]. The proportion of antimicrobial resistance was 58.3% in Nigeria, 12.5% in Ghana, 12.5% in Senegal [13], and more than half (60%) were from East Africa [14]. The prevalence of resistance to penicillin among *Streptococcus pneumoniae* isolates ranged from 25–100% and 1.3–60% among tested isolates for extended-spectrum beta-lactamase reported in sub-Saharan Africa [15].

A systematic review and meta-analysis study conducted in Ethiopia reported, nearly half of the patients (48.5%) took antibiotics. Of those participants, more than 49% used antibiotics inappropriately and 59.7% resistance strains were identified. This study also showed practicing non-accordance to guidelines in the management of infectious diseases is a big challenging problem in Ethiopia which makes many bacteria resistant to antibiotics [16]. Therefore, the best way to tackle the consequence of antibiotics misuse will rely on the preservation of the existing antibiotics and designing appropriate antibiotics use strategies [17]. If irrational antibiotic utilization is not tackled early in health facilities, their consequences can be a serious problem to human beings near future [16, 18, 19].

Although different cross-sectional studies were conducted at community and outpatient levels on assessment of antimicrobial-related problems and their costs; antibiotics and their resistance patterns; and challenges of antibiotics use [20–23], there are limited studies addressing the antibiotic utilization at inpatient setting in Ethiopia, particularly at the study area. Therefore, this study aimed to assess the antibiotics use among hospitalized adult patients in Jimma Medical Center (JMC), southwestern Ethiopia, using a prospective observational study design.

Given the above problems and knowing the decline in the development of new antibiotics to combat the problems, it is important to investigate the antibiotics use pattern among hospitalized patients in Jimma Medical Center, to pave the way for antimicrobial stewardship using available antibiotics and resources on hand. Thus, this study will serve as input for the health care systems, health professionals, hospitalized patients, governmental and non-governmental organizations (NGOs), and ministers of health to make evidence-based decisions in the study area and other similar hospitals by large.

## Methods and participants

### Study design, study settings and period

A hospital-based prospective observational study conducted at the Internal Medicine, Surgery, and Gynecology/Obstetrics wards of Jimma Medical Center (JMC). JMC is the only tertiary hospital with a bed capacity of 800 in the southwestern part of Ethiopia located in Jimma town, 352 km far from Addis Ababa. The hospital has four major wards (Internal Medicine, Surgery, Gynecology/Obstetrics, and Pediatrics) and five other departments. It provides services for approximately 15,000 inpatients and 160,000 outpatient clients per year with a catchment population of about 20 million people living within the wide catchment area of the Jimma zone and the surrounding. It can serve 11,000 emergency cases and 4500 deliveries per year, and it has 1600 staff members with 32 care units. The study was conducted from 30 October 2020 to 29 January 2021.

### Population

All adult patients who were admitted to Internal Medicine, Surgery, and Gynecology/Obstetrics wards of JMC; and who were taking antibiotics for treatment and/or prophylaxis purposes were source population. All hospitalized adult patients at Internal Medicine, Surgery, and Gynecology/Obstetrics wards of JMC, who were fulfilled the inclusion criteria during the study period were study population for this study.

### Eligibility criteria

All adult patients who were admitted to Internal Medicine, Surgery, and Gynecology/Obstetrics wards and who received at least one systemic antibiotic for treatment and/or prophylaxis during the study period were included in the study. However, all patients with age less than 18 years; all adult patients who refused to participate in the study; and patients who were taking only anti-mycobacterial agents were excluded from the study. Note: Anti-mycobacterial agents are four drugs in fixed dose combination. It is difficult to compare them with a single antibiotic and calculating days of therapy for them can overestimate the study results as they taken throughout study period and beyond.

### Sample size and sampling technique

Sample size (*n*) was calculated by using a single population proportion formula:

$$n= {(Z\alpha /2)}^{2}* \frac{P\left(1-P\right)}{{w}^{2}}={\left(1.96\right)}^{2}* \frac{0.50\left(1-0.50\right)}{{0.05}^{2}}=384,$$

where ‘*P*’ is the estimated proportion of antibiotic use practice in Internal Medicine, Surgery, and Gynecology/Obstetrics wards, which is 50% (*P* = 0.5). ‘*Z*’ is level of confidence = 1.96 with 95% confidence interval; and ‘*W*’ is the margin of error that the investigators are willing to accept, which is 5%.

Since *N* is less than 10,000 (*N* = 2174 patients: 918, 640 and 616 patients were from Internal Medicine, Surgery, and 616 Gynecology/Obstetrics ward, respectively), taken from three wards 1 year before the study period at a similar period, from 30 October 2019 to 29 January 2020. The information was obtained from the health management information system of the JMC report (hospital statistics office). By using the correction formula:

$$\mathrm{nf}=\frac{n}{\begin{array}{c}1+\frac{n}{N}\\ .\end{array}} =\frac{384}{\begin{array}{c}1+\frac{384}{2174}\\ .\end{array}}=327.$$

After adding 10% for non-response rate, the final sample size of this study was 360 adult admitted patients. Consecutive patients were recruited and consecutive sampling technique was used to collect data from 360 adult hospitalized patients from Internal Medicine (*n* = 152, 42.2%), Surgery (*n* = 106, 29.4%), and Gynecology/Obstetrics (*n* = 102, 28.3%), wards of JMC. Patients were recruited from each ward based on the reports of proportion of patient flow to each ward 1 year before the study period, from 30 October 2019 to 29 January 2020.

### Data collection procedures

A semi-structured data abstraction format was developed from different studies. After the pre-test, the corrected version of the questionnaire was used to collect data. Data were collected through medical record reviews and patient interviews. The data collection involved three clinical pharmacists of Jimma Medical Center.

### Data quality management

To maximize the quality of the data, training was given for data collectors for 1 day on how to fill the data collection instruments and extract the necessary information. The investigator also did close supervision and monitoring weekly and the whole quality of data was assured that all the facilities were measured in the same way in all the time considered. In addition, a pre-test was conducted using 5% of the total sample size (18 patients), outside of the study period. Patient medical records, admission, and discharge charts were used to collect data and patients were also interviewed for subjective data that were not recorded in patient’s medical records. After data collection, data were cleared, categorized, compiled, and checked for completeness and accuracy before being analyzed. Any erroneous and ambiguous data were excluded.

### Data analysis procedures

The collected data were coded, entered into Epi-data, and exported to statistical package for social science (SPSS^®^) version 23.0 software for analysis. Categorical and continuous data were expressed as percentages and mean ± SD, respectively, to summarize patient baseline characteristics and the study findings.

Utilization rates were presented as days of therapy (DOT) per 100(0) bed-days (BD) to compare rates over time or among different wards and risk group participants. The most commonly used denominator to capture time at risk for patients was BD. Bed-days was calculated by an SPSS that measures the total number of occupied beds on inpatient wards per calendar day. Similarly, the DOT was also calculated and defined as a day of therapy as any calendar day in which at least a single dose of antibiotic was received. DOTs were counted separately for each antibiotic agent (for example, a patient on two different antibiotics simultaneously would count as 2 DOT on a single calendar day). World Health Organization Essential Medicine List (WHO 2019) and Ethiopian Essential Medicine List (EEML 2020) “Access, Watch, and Reserve (AWaRe)” antibiotics classification were used to assess antibiotic use pattern among participants.

## Results

### Socio-demographic and disease characteristics of patients

Females accounted for about 55.3% of the study participants and 29.2% were housewives. The mean age (SD) of participants was 37.65 ± 16.75 (range: 18–99), and most of them, 262 (72.8%), were between the age range of 18 and 47 years. The majority of the study participants were attended formal education (59.4%), living in rural areas (61.4%), and farmers (24.4%). More than one-third of the participants had not a regular monthly income (Table 1).

**Table 1:** Socio-demographic characteristics of hospitalized adult patients in JMC from 30 October 2020 to 29 January 2021 (*n* = 360)

Socio-demographic characteristics of patients	Wards of Jimma Medical Center (JMC)			Total, *N* (%)
	Medical, *N* (%)	Surgical, *N* (%)	GYN/OBS, *N* (%)	
WHO standard age group **(**years)				
Young group (18–47)	96 (26.7)	72 (20.0)	94 (26.1)	262 (72.8)
Middle age (48–63)	38 (10.6)	17 (4.7)	7 (1.9)	62 (17.2)
Elderly (64 and above)	18 (5.0)	17 (4.7)	1 (0.3)	36 (10.0)
Average age (mean ± SD) (*R*)	41.0 ± 18.2 (18–99)	41.1 ± 17.0 (18–80)	29.0 ± 9.8 (18–65)	37.7 ± 16.8 (18–99)
Sex of patient				
Male	84 (23.3)	77 (21.4)	NA	161 (44.7)
Female	68 (18.9)	29 (8.1)	102 (28.3)	199 (55.3)
Residence areas				
Urban	51 (14.2)	37 (10.3)	51 (14.2)	139 (38.6)
Rural	101 (28.1)	69 (19.2)	51 (14.2)	221 (61.4)
Smoking status				
Non-smokers	125 (34.7)	96 (26.7)	95 (26.4)	316 (87.8)
Smokers	27 (7.5)	10 (2.8)	7 (1.9)	44 (12.2)
Education status				
No formal education	77 (21.4)	34 (9.4)	35 (9.7)	146 (40.6)
Primary education	51 (14.2)	60 (16.7)	53 (14.7)	164 (45.5)
Secondary and above	15 (6.7)	12 (3.3)	614(3.9%)	50 (13.9)
Marital status				
Single	29 (8.1)	27 (7.5)	8 (2.2)	64 (17.8)
Married	97 (26.9)	69 (19.2)	86 (23.9)	252 (70.0)
Divorced	10 (2.8)	6 (1.7)	3 (0.8)	19 (5.3)
Widowed	16 (4.4)	4 (1.1)	5 (1.4)	25 (6.9)
Occupation				
Employee	15 (4.2)	11 (3.1)	12 (3.3)	38 (10.6)
Merchant	21 (5.8)	20 (5.6)	10 (2.8)	51 (14.2)
Housewife	39 (10.8)	16 (4.4)	50 (13.9)	105 (29.2)
Farmer	42 (11.7)	36 (10.0)	10 (2.8)	88 (24.4)
Student	25 (6.9)	17 (4.7)	13 (3.6)	55 (15.3)
Daily labor	10 (2.8)	6 (1.7)	7 (1.9)	23 (6.4)

Of all the infection diagnoses, pneumonia (16.1%), urinary tract infection (15.3%), and acute appendicitis (13.9%) were the most common infectious diagnoses followed by traumatic injuries (7.8%), sepsis (7.5%), meningitis (6.7%) and opportunistic infections (5.0%). Pneumonia (14.2%), acute appendicitis (12.8%), and sepsis (5.8%) were the common infections in Internal Medicine, Surgery, and Gynecology/Obstetrics ward, respectively (Table 2).

**Table 2:** Infection diagnosis and types of suspected infectious condition among hospitalized adult patients in JMC wards during the study period (*n* = 360)

Infection diagnosis (ICD 10)*	Wards of JMC			Total *N* (%)
	Medical, *N* (%)	Surgical, *N* (%)	GYN/OBS, *N* (%)	
Pneumonia	52 (14.2)	2 (0.6)	4 (1.1)	58 (16.1)
Abscess	5 (1.4)	10 (2.8)	1 (0.3)	16 (4.4)
Acute appendicitis	0 (0.0)	46 (12.8)	4 (1.1)	50 (13.9)
Acute cholangitis	0 (0.0)	5 (1.4)	0 (0.0)	5 (1.4)
Diabetic foot ulcer	6 (1.7)	0 (0.0)	0 (0.0)	6 (1.7)
Gastroenteritis	11 (3.1)	0 (0.0)	0 (0.0)	11 (3.1)
Hospital acquire infections	7 (1.9)	0 (0.0)	0 (0.0)	7 (1.9)
Infective endocarditis	4 (1.1)	0 (0.0)	1 (0.3)	5 (1.4)
Meningitis	24 (6.7)	0 (0.0)	0 (0.0)	24 (6.7)
Nephrotic syndromes	5 (1.4)	1 (0.3)	3 (0.8)	9 (2.5)
Fever of neutropenia	5 (1.4)	0 (0.0)	0 (0.0)	5 (1.4)
Opportunistic infections**	18 (5.0)	0 (0.0)	2 (0.6)	20 (5.6)
Parapneumonic effusion/empyema	9 (2.5)	4 (1.1)	0 (0.0)	13 (3.6)
Sepsis	6 (1.7)	0 (0.0)	21 (5.8)	27 (7.5)
Sexually transmitted infections	2 (0.6)	0 (0.0)	1 (0.3)	3 (0.8)
Skin and soft tissue infections	5 (1.4)	3 (0.8)	0 (0.0)	8 (2.2)
Spontaneous bacterial peritonitis	9 (2.5)	0 (0.0)	0 (0.0)	9 (2.5)
Surgical site infections	0 (0.0)	12 (3.3)	8 (2.2)	20 (5.6)
Traumatic injuries	0 (0.0)	20 (5.6)	8 (2.2)	28 (7.8)
Urinary tract infection	31 (8.6)	10 (2.3)	14 (3.9)	55 (15.3)
Other infections***	3 (0.8)	2 (0.6)	2 (0.6)	7 (1.9)

^*^A given patient may have > 1 diagnosis

**Pneumocystis pneumonia [13], and Toxoplasmosis [7]

***Symptomatic myoma [1], chronic diarrhea [2], and unknown infections [4]

More than half of study participants 196 (54.4%) had comorbidity conditions. Of all the comorbidities, hypertension 38 (19.4%), tuberculosis 17 (18.9%), and heart failure 35 (17.9%) were the most common comorbidities and followed by acute/chronic kidney diseases 26 (13.3%) and 26 (13.3%) chronic pulmonary diseases (Table 3).

**Table 3:** Infections and non-infectious comorbidities among hospitalized adult patients in JMC wards during the study period (*n* = 360)

Comorbidities and risk status indicators	Wards of JMC			Total, *N* (%)
	Medical, *N* (%)	Surgical (%)	GYN/OBS (*N*)	
Presentence of comorbidities				
No	16 (4.4)	81 (22.5)	67 (18.6)	164 (45.6)
Yes	136 (37.8)	25 (6.9)	35 (9.7)	196 (54.4)
Total	152 (42.2)	106 (29.5)	102 (28.3)	360 (100)
Infections and non-infectious comorbidities*				
Hypertension	23 (11.7)	7 (3.6)	8 (4.1)	38 (19.4)
Heart failure	29 (14.8)	1 (0.5)	5 (2.6)	35 (17.9)
Diabetes mellitus	15 (7.7)	2 (1.0)	3 (1.5)	20 (10.2)
Acute/chronic kidney diseases	25 (12.8)	1 (0.5)	0 (0.0)	26 (13.3)
Moderate/severe liver diseases	10 (5.1)	0 (0.0)	0 (0.0)	10 (5.1)
Neurologic diseases	4 (2%)	3 (1.5)	1 (0.5)	8 (4.1%)
Tuberculosis	34 (17.3)	3 (1.5)	0 (0.0)	37 (18.9)
HIV/AIDS	15 (7.7)	0 (0.0)	2 (1.0)	17 (8.7)
Metastatic solid tumor	8 (4.1)	6 (3.1)	10 (5.1)	24 (12.2)
Other malignancies**	9 (4.6)	1 (0.5)	4 (2.0)	14 (7.1)
Asthma and COPD	16 (8.2)	4 (2%)	6 (3.1)	26 (13.3)
Neurologic diseases	4 (2%)	3 (1.5)	1 (0.5)	8 (4.1%)
Anemia of chronic diseases	6 (3.1)	4 (2%)	0 (0.0)	10 (5.1)
Malaria	3 (1.5)	0 (0.0)	0 (0.0)	3 (1.5)
Esophageal candidiasis	1 (0.5)	1 (0.5)	0 (0.0)	2 (1.0)

^*^A given patient may have > 1 comorbidities

^**^Hematological malignancies and non-metastatic solid tumor

### Antibiotics utilization patterns

**Fig. 1:**
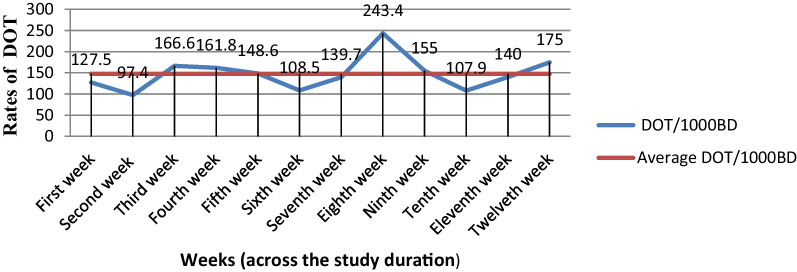
Trends of antibiotics consumption at JMC wards among adult hospitalized patients in DOT/1000BD during the study duration (*n* = 360)

**Fig. 2:**
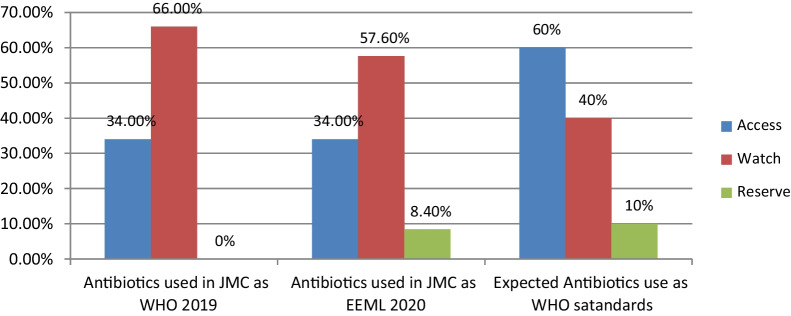
Antibiotics use patterns in JMC wards according to Essential Medicine List (EML) AWaRe classification as compared to WHO standards (*n* = 360)

A. Antibiotics use patterns using antibiotics metrics
  A given patient with an infection diagnosis was exposed to 1–10 antibiotics with a mean of greater than two. The mean DOT per patient was 21.5, 12.8, and 8.9 for medical, surgical, and gynecology/obstetrics wards, respectively. The mean of DOT per patient was 15.4 ± 14.4 SD with an overall average (mean ± SD) bed-days of 13.9 ± 9.0 for the settings.
  The overall rate of antibiotics consumption during the study was 111 per 100 BD. The consumption of antibiotics was 120.9 DOT/100 BD in Internal Medicine, 105.1 DOT/100 BD in Surgery, and 91.8 DOT/100 BD in the Gynecology/Obstetrics ward. The frequency of daily antibiotics consumption during the study period was higher in the Medical ward 3266 DOTs (61.1%) compared with surgery ward 1359 DOTs (24.5%) versus 912 DOTs (16.4%) in the Gynecology/Obstetrics ward, relative to total DOTs. For frequently prescribed antibiotics, the percentage of consumption for cephalosporins, nitro-imidazole, glycopeptides, penicillins, trimethoprim–sulfonamide, and fluoroquinolones were 5982 DOTs (108%), 2699 DOTs (48.7%), 1970 DOTs (35.6%), 766 DOTs (13.8%), 646 DOTs (11.7%) and 632 DOTs (11.4%), respectively, relative to total DOTs (Table 4).
  The trends of DOT/1000BD over time were shown a small decrease in the 1st, 5th, and 10th week and an increase in the 2nd, 6th and last 2 weeks. But a similar pattern was seen during the 3rd and 4th week, followed by a sharp increase between the 6th and 10th week of the study duration (Fig. 1).
B. Antibiotics use patterns based on EML AWaRe classification
  Cephalosporins were the most commonly used class of antibiotics (93.9%). The most commonly used specific antibiotics in JMC were ceftriaxone (86.7%), Metronidazole (38.6%), vancomycin (15%), and azithromycin (8.6%) across the study duration (Table 5). The indicator level of antibiotics use for “Access” group antibiotics was 34% in this study based on both the World Health Organization (WHO 2019) Essential Medicine List (EML) and the Ethiopian Essential Medicines List (EEML 2020) recommendations (Fig. 2).
  In this study, two-thirds (66%) of the antibiotics used in JMC wards were from the “Watch” group as WHO 2019 EML AWaRe groups and more than half (57.6%) based on EEML 2020 AWaRe Classification. The setting has not had “Reserve” group antibiotics as WHO 2019 EML AWaRe Classification despite the high use of “Watch” groups. But 8.4% of “Reserve” group antibiotics were used in JMC as EEML 2020 AWaRe Classification (Table 5).

**Table 4:** The rates of antibiotics consumption by ward types, patient demographics, the purpose of use, and types of antibiotics used among hospitalized adult patients at JMC (*n* = 360)

Variables	Sum of DOT (%)	Sum of BD	DOT/100BD
Wards JMC			
Medical	3266 (61.1)	2702	120.9
Surgical	1359 (24.5)	1293	105.1
GYN/OBS	912 (16.4)	993	91.8
Sex of the patients			
Male	3024 (54.6)	2437	124.1
Female	2513 (45.4)	2551	98.5
WHO standard age group			
18–47 years old (young group)	4021 (72.6)	3454	116.4
48–63 years old (middle age group)	1021 (18.4)	1022	99.9
≥ 64 years old (elderly group)	495 (9.0)	512	96.7
Antibiotics use purposes			
Prophylaxis	319 (5.8)	703	45.4
Treatment	3814 (68.9)	3243	117.6
Both prophylaxis and treatment	1404 (25.3)	1042	134.7

Rates of antibiotics utilization in JMC during the study duration			
Overall days of therapy and bed-days (denominators)	5537 (100.0)	4988	111DOT/100BD
Penicillins			
Ampicillin, amoxicillin, and cloxacillin	766 (13.8)	The common denominator (4988)	15.36
Cephalosporins			
Cephalexin, ceftriaxone, and ceftazidime	5982 (108)		119.93
Macrolides			
Azithromycin and clarithromycin	508 (9.2)		10.18
Fluoroquinolones			
Ciprofloxacin and norfloxacin	632 (11.4)		12.67
Augmentin			
Amoxicillin–clavulanic acid	130 (2.3)		2.61
Cotrimoxazole			
Trimethoprim–sulfonamide	646 (11.7)		12.95
Tetracyclines			
Doxycycline	284 (5.1)		5.69
Aminoglycosides			
Gentamicin	84 (1.5)		1.68
Nitro-imidazole			
Metronidazole	2699 (48.7)		54.11
Carbapenems			
Meropenem	111 (2.0)		2.23
Glycopeptides			
Vancomycin	1970 (35.6)		49.49
Others			
Other antibiotics*	174 (3.1)		3.48
Mean ± standard deviation (Range)	15.4 ± 14.4 (1–90)	13.9 ± 9.0 (1–67)	

^*^Benzathine penicillin and erythromycin

**Table 5:** Class of antibiotics and percentage of antibiotics used among hospitalized patients in JMC during the study period based on EML AWaRe classification (*n* = 360)

S.N	ATC code	Antibiotics class	Antibiotics	Total, *N* (%)	WHO 2019 AWaRe group	EEML 2020 AWaRe group	Antibiotics use (%)	
							WHO 2019	EEML 2020
1.	J01CA01	Penicillins	Ampicillin	11 (3.1)	Access	Access	34.0%	34.0%
2.	J01CA04	Penicillins	Amoxicillin	5 (1.4)	Access	Access		
3.	J01CR02	Amoxicillin–clavulanic acid	Augmentin	5 (1.4)	Access	Access		
4.	J01DB	Cephalosporin	Cephalexin	26 (7.2)	Access	Access		
5.	J01CF	Penicillins	Cloxacillin	1 (0.3)	Access	Access		
6.	J01EE01	Trimethoprim–sulfonamide	Cotrimoxazole	11 (3.1)	Access	Access		
7.	J01AA02	Tetracyclines	Doxycycline	22 (6.1)	Access	Access		
8.	J01GB03	Aminoglycosides	Gentamicin	3 (0.8)	Access	Access		
9.	J02AB01	Nitro-imidazole	Metronidazole	139 (38.6)	Access	Access		
10.	J01MA	Fluoroquinolones	Norfloxacin	3 (0.8)	Access	Access		
11.	J01XX	Other antibiotics*	Others	9 (2.5)	Access	Access		
12.	J01FA10	Macrolides	Azithromycin	31 (8.6)	Watch	Watch		
13.	J01DD04	Cephalosporin	Ceftriaxone	312 (86.7)	Watch	Watch	66.0%	57.6%
14.	J01DD02	Cephalosporin	Ceftazidime	25 (6.9)	Watch	Watch		
15.	J01FA09	Macrolides	Clarithromycin	1 (0.3)	Watch	Watch		
16.	J01MA02	Fluoroquinolones	Ciprofloxacin	29 (8.1)	Watch	Watch		
17.	J01DH02	Carbapenems	Meropenem	4 (1.1)	Watch	Reserve		8.4%
18.	J01XA01	Glycopeptides	Vancomycin	54 (15.0)	Watch	Reserve		
		Total number of antibiotics		691 (100)			691 (100.0)	691 (100.0)

*ATC code* The Anatomical Therapeutic Chemical code for antibiotics

*Benzathine penicillin and erythromycin

## Discussion

Excessive antibiotics exposure lead to a complex relationship between antibiotic resistance and irrational antibiotics use [24]. Thus, knowing the extent of antibiotics exposure, patterns of antibiotic use and identifying risk groups can be helpful to prevent the occurrence of inappropriate antibiotics usage and its consequences. A given adult hospitalized patient with a bacterial infection diagnosis was exposed to one to ten antibiotics with a mean of greater than two in study setting. This finding is almost similar with study conducted in different Referral Hospitals of Ethiopia [16, 25, 26].

In this study, the consumption rates of antibiotics were 120.9 DOT/100BD in Internal Medicine, 105.1 DOT/100BD in Surgery, and 91.8 DOT/100BD in Gynecology/Obstetrics ward. The difference between wards indicates there was high antibiotics exposure (DOT) in the medical and surgical ward with equal risk to be exposed (bed-days). The overall rate of antibiotics consumption was 111/100BD across study duration. This finding is in line with the study conducted in Belgium, which showed 1232 DOT/1000BD [27]. But result of current study is higher than reports from different parts of the USA [28, 29] and reports from California that reported 436–509 DOT/1000BD [30]; and lower than a study done in Ethiopia, which reported 1549 DOT/1000BD [22]. This discrepancy might be due to differences in time, study designs, settings, and treatment modalities or/and prophylaxis protocol used in hospitals.

Based on the present study, the indicator level of “Access” group antibiotics was 34% in a setting which is too low compared to WHO Essential Medicine List AWaRe Classification standard, at least 60% of institutional antibiotics consumption [2]. This finding is in line with the global point prevalence survey conducted in 69 countries in 664 hospitals that resulted in the regional “Access” antibiotics use ranged from 28.4% in West and Central Asia to 57.7% in Oceania [31], and 34.5% in four developing countries [32].

Rapid increases in the “Watch” group antibiotic use, particularly in developing countries like Ethiopia, are a big challenge in antimicrobial stewardship [10]. The current study showed that nearly two-thirds (66%) of antibiotics used were from the “Watch” group antibiotics. This finding is comparable to the survey that reported 66.1% in West and Central Asia [31], and 64.4% in four low and middle-income countries [32]. High consumption of third-generation cephalosporins such as ceftriaxone (86.7%) in the study area might be a possible reason for the high volume of their use. Thus, this high antibiotics consumption needs the implementation of ASP in health facilities to ensure rational “Watch” group antibiotic use [12].

This study indicated the commonly prescribed antibiotics were ceftriaxone (86.7%), metronidazole (38.6%), vancomycin (15%), and azithromycin (8.6%) based on the frequency of specific drugs used among study participants. Cephalosporins were the most commonly used class of antibiotics (93.9%). In comparison, the study from northern Nigeria reported that cephalosporins represented one-third of antibiotics used [33], and this class of antibiotics accounted for 67.2% in Lahore, Pakistan [34], and they are highly consumed in different parts of Ethiopia [16, 25, 26, 35]. The extensive use of third-generation cephalosporins like ceftriaxone has been a focus of study in Ethiopian health facilities including JMC [25, 26, 36].

## Limitations of the study

Unlike the previous works, this study was prospective observational study. This makes the study a timely, relevant and comprehensive in the investigation of the facts regarding antibiotics utilization patterns among adult hospitalized patients. Despite it was a prospective study, this study had several limitations. First, it was conducted in a single hospital. Practice patterns and antibiotics resistance patterns may vary among hospitals and so, this may limit generalizability of the study. Second, most of the diagnoses were clinical and cultures were not accessible most of the time. Third, this study assess the trends of antibiotics use among hospitalized adult patients by descriptive statistics and did not address the factors that could be related to the increase in antibiotic use in hospitals. Finally, there was limited data that was conducted using a similar study design to compare the findings.

## Conclusions

A higher antibiotic exposure and consumption was frequently observed among adult hospitalized patients in the study area. There was a rapid increase in “Watch” group antibiotics use and about two-thirds of the prescribed antibiotics were from this group. The third-generation cephalosporins were the most commonly used class of antibiotics. Generally, this study will provide information on antibiotics utilization patterns among hospitalized adult patients in Jimma Medical Center, Southwestern Ethiopia, and identify targets (“Watch” and “Reserve” group antibiotics) for better antibiotics utilization in antimicrobial stewardships that prevent antibiotics misuse in health facilities. It will also serve as an insight for the development of hospital guidelines, essential medicines lists, and formularies in study area and beyond.

## Data Availability

The data used in this study can be accessible upon request from the corresponding author.

## Abbreviations

AMR: Antimicrobial resistance
ASP: Antimicrobial Stewardship Programs
BD: Bed-days
DOT: Days of therapy
EML: Essential Medicine List
EEML: Ethiopian Essential Medicine List
GYN/OBS: Gynecology/Obstetrics
JMC: Jimma Medical Center
WHO: Word Health Organization
